# Influence of Yttrium on the Thermal Stability of Ti-Al-N Thin Films

**DOI:** 10.3390/ma3031573

**Published:** 2010-03-04

**Authors:** Martin Moser, Daniel Kiener, Christina Scheu, Paul H. Mayrhofer

**Affiliations:** 1 Department of Physical Metallurgy and Materials Testing, Montanuniversität Leoben, Franz Josef Strasse 18, 8700 Leoben, Austria; E-Mail: martin.moser@ruag.com (M.M); 2 Department of Chemistry, Ludwig-Maximilians-University Munich, Butenandtstr. 5-13 (E), 81377 Munich, Germany; E-Mails: daniel.kiener@unileoben.ac.at (D.K.); christina.scheu@cup.uni-muenchen.de (C.S.)

**Keywords:** TiAlN, Yttrium, thermal stability, decomposition

## Abstract

Ti_1-x_Al_x_N coated tools are commonly used in high-speed machining, where the cutting edge of an end-mill or insert is exposed to temperatures up to 1100 °C. Here, we investigate the effect of Yttrium addition on the thermal stability of Ti_1-x_Al_x_N coatings. Reactive DC magnetron sputtering of powder metallurgically prepared Ti_0.50_Al_0.50_, Ti_0.49_Al_0.49_Y_0.02_, and Ti_0.46_Al_0.46_Y_0.08_ targets result in the formation of single-phase cubic (c) Ti_0.45_Al_0.55_N, binary cubic/wurtzite c/w-Ti_0.41_Al_0.57_Y_0.02_N and singe-phase w-Ti_0.38_Al_0.54_Y_0.08_N coatings. Using pulsed DC reactive magnetron sputtering for the Ti_0.49_Al_0.49_Y_0.02_ target allows preparing single-phase c-Ti_0.46_Al_0.52_Y_0.02_N coatings. By employing thermal analyses in combination with X-ray diffraction and transmission electron microscopy investigations of as deposited and annealed (in He atmosphere) samples, we revealed that Y effectively retards the decomposition of the Ti_1-x-y_Al_x_Y_y_N solid-solution to higher temperatures and promotes the precipitation of c-TiN, c-YN, and w-AlN. Due to their different microstructure and morphology already in the as deposited state, the hardness of the coatings decreases from ~35 to 22 GPa with increasing Y-content and increasing wurtzite phase fraction. Highest peak hardness of ~38 GPa is obtained for the Y-free c-Ti_0.45_Al_0.55_N coating after annealing at T_a_ = 950 °C, due to spinodal decomposition. After annealing above 1000 °C the highest hardness is obtained for the 2 mol % YN containing c-Ti_0.46_Al_0.52_Y_0.02_N coating with ~29 and 28 GPa for T_a_ = 1150 and 1200 °C, respectively.

## 1. Introduction

Owing to their excellent physical, chemical, and mechanical properties, Ti_1-x_Al_x_N coatings are used in a broad field of applications, ranging from the protection of components in aerospace and the automotive industry to the utilization as hard and wear resistant overlays on e.g., tools, dies, and moulds [1,2,3]. In high-speed machining, where the cutting edge of an end-mill or insert is exposed to temperatures up to 1100 °C, Ti_1-x_Al_x_N coated tools are commonly employed [4]. For these applications, the oxidation resistance, thermal stability and microstructure are crucial in influencing the tool life time and performance [5].

When grown by plasma-assisted vapor deposition techniques Ti_1-x_Al_x_N crystallizes in a supersaturated solid-solution cubic (c) NaCl structure up to AlN mole fractions x ~0.7. This value strongly depends on the deposition-conditions used and the resulting defect-density, microstructure, and stress state of the coatings [6,7]. Hence, various maximum AlN fractions in single-phase c-Ti_1-x_Al_x_N coatings are reported in the literature. Exceeding the metastable AlN solubility results in a mixed (NaCl + ZnS-wurtzite) structure, or the films crystallize completely in the ZnS-wurtzite (w) modification [6,7,8,9,10,11,12,13]. As c-Ti_1-x_Al_x_N films with high Al contents have superior physical, mechanical, and chemical properties, and show better oxidation resistance than their two-phased or single-phased wurtzite counterparts, they are generally preferred for industrial applications.

The thermal stability and the microstructural evolution during thermal exposure of advanced hard coatings can be seen as key factors for their optimal performance when used on tools or components that are exposed to high temperatures. Therefore, the investigation of the thermal stability of such coatings is of high interest [14]. In recent years, combined thermal analyses were used to *in situ* derive reaction temperatures, energy inputs and associated mass changes through the formation of reaction products or volatile species [11,15,16,17,18]. The decomposition process of Ti_1-x_Al_x_N has been described in detail for AlN mole fractions x ranging from 0.25 to 0.75 [2,9,11,13,16,19,20,21,22].

Further improvement of the coating properties and thermal stability can be realized by alloying with beneficial elements, e.g., reactive elements [5,23]. The element yttrium is used to improve oxidation resistance of structural and coating materials [24,25]. However, it is important to consider that especially metastable phases are sensitive to any chemical variation. Recently, we showed by *ab initio* calculations that the addition of 6.75 at % Y to the metal sublattice of Ti_1-x_Al_x_N decreases the metastable solubility limit for AlN in cubic Ti_1-x_Al_x_N from x_c-max_ ~0.67 to 0.50 [26]. The *ab initio*-derived results were verified by experimental studies exhibiting a single-phase cubic structure for Ti_0.45_Al_0.55_N, a mixed cubic/wurtzite structure for Ti_0.41_Al_0.57_Y_0.02_N and Ti_0.43_Al_0.52_Y_0.05_N, and a single-phase wurtzite structure for Ti_0.38_Al_0.54_Y_0.08_N. Due to the formation of a wurtzite phase in these Y-containing Ti_1-x_Al_x_N films their hardness is ~22 GPa, much smaller than the ~35 GPa obtained for the Y-free c-Ti_0.45_Al_0.55_N film [26]. By optimizing the deposition conditions when using the Ti_0.49_Al_0.49_Y_0.02_ target, coatings can be prepared exhibiting a single-phase cubic structure with a chemical composition of c-Ti_0.46_Al_0.52_Y_0.02_N and a hardness of ~33 GPa [27]. As described in [27], the optimized deposition conditions result in an increased energy transferred to the growing species as well as a changed chemical composition of the growing film towards lower Al contents. Hence, the coating composition shifts deeper into the metastable cubic phase field.

In the present study, we investigate the thermal decomposition process of Ti_1-x-y_Al_x_Y_y_N films having YN contents of 0, 2, and 8 mol %. We applied electron probe microanalyses, X-ray diffraction (XRD), and transmission electron microscopy (TEM) for chemical and structural investigations of as deposited and annealed samples. The decomposition process itself is monitored by simultaneous thermal analyses (STA), combining differential scanning calorimetry (DSC), thermo-gravimetric analyses (TGA), and mass spectrometry (MS). These investigations allow for a correlation of hardness variations with microstructure and phase-composition variations as a function of the thermal treatment.

## 2. Experimental Section

Ti_1-x-y_Al_x_Y_y_N thin films were grown on MgO (100) and Si (100) substrates (20 × 7 × 0.35 mm³) and low-alloyed steel foils in a modified Leybold Z-400 facility by reactive unbalanced magnetron sputtering from a Ø76 mm Ti_0.50_Al_0.50_, Ti_0.49_Al_0.49_Y_0.02_, and Ti_0.46_Al_0.46_Y_0.08_ compound target (powder-metallurgically prepared by PLANSEE GmbH, Lechbruck, Germany, 99.9% purity) in a mixed Ar-N_2_ (both with 99.999% purity) glow discharge using a N_2_/Ar partial pressure ratio of 0.4 and a working gas pressure of 0.5 Pa. The substrate temperature of 550 °C and DC bias potential of -50 V were kept constant for all deposition runs. The substrates were positioned 5 cm above the target race track (substrates are parallel aligned to the target surface) as schematically shown in [27]. An ENI RPG-50 asymmetric bi-polar pulsed power supply, set to provide 400 W in power regulation mode, was used for DC and for pulsed DC magnetron sputtering. The pulsing frequency of a +37 V reverse voltage pulse was set to 80 kHz and the positive pulse length *t*_rev_ was kept constant at 4976 ns to guarantee a single-phase cubic growth of a Ti_0.46_Al_0.52_Y_0.02_N film (*i.e.*, with 2 mol % YN), for more details see [27].

Simultaneous thermal analyses in the temperature range between 450 and 1500 °C were conducted using a Netzsch STA 409 instrument. The measurements were carried out with a heating rate of 20 K/min in flowing He atmosphere (99.9% purity, 20 sccm flow rate). Post-deposition annealing to distinct temperatures T_a_ was performed in He atmosphere using a heating rate of 20 K/min immediately followed by a cooling step at a rate of 40 K/min.

The structure and phase identification of the thin films were investigated using a Siemens D500 X-ray diffractometer with Cu-Kα radiation in the 2θ range 30−65 deg in Bragg-Brentano geometry. For the classification of the obtained reflexes, the JCPDS database was used [29]. The 2θ positions of the solid-solution cubic and wurtzite Ti_0.5_Al_0.5_N phases are derived from lattice constants obtained by *ab initio* calculation, see [26].

To avoid overlap with the substrate material contributions, thermal analyses and annealing experiments for XRD were performed on powdered film material after chemical removal from their low alloy steel substrates with 10 mol % nitric acid. TEM plan view samples were also prepared from free-standing film material, which was subsequently ion-thinned to electron-microscopy transparency using a Gatan PIPS. TEM measurements were conducted on a Philips CM12 at 120 keV to study the coating morphology in the as deposited state and after annealing. Furthermore, selected coatings were investigated by high resolution TEM (HRTEM) and high angle annular dark field (HAADF) imaging in scanning TEM mode using a FEI Titan 80–300 keV. This microscope is equipped with an energy dispersive X-ray detector from EDAX and a post-column energy filter (GIF Tridiem from Gatan) for analytical investigations, which were also performed at selected coatings. The Titan was operated either at 80 keV or 300 keV.

During scanning electron microscopy (SEM) investigations with a Zeiss EVO 50 microscope, the average chemical composition of the films was determined by energy dispersive x-ray analysis (EDX) with an Oxford Instruments INCA EDX unit using metallic Al and Y and a TiN film standard. Quantification of the latter was obtained by Rutherford backscattering spectroscopy as described in [30].

The hardness (H) of coatings deposited on MgO in their as deposited state and after annealing at different T_a_ in He was measured by nano-indentation with a CSIRO ultra micro indentation system (UMIS) equipped with a Berkovich-indenter. With respect to a proper statistic at least 50 indents are performed for each sample with maximum loads ranging from 10 to 45 mN keeping the indentation depth below 10% of the coating thickness, which was ~3.0 µm for all coatings investigated.

## 3. Results and Discussion

EDX analyses in the SEM yielded N contents of 51.0 ± 0.5 at % and O contents below the detection limit for the coatings investigated. The Y-free DC magnetron sputtered reference coating had an elemental composition in the metallic sub-lattice of Ti = 45 at % and Al = 55 at % and a single-phase cubic microstructure after deposition. This film will be referenced throughout the work as c-Ti_0.45_Al_0.55_N. Using the same standard conditions during DC sputtering, and equipping the magnetron with a Ti_0.49_Al_0.49_Y_0.02_ target, resulted in the formation of a binary phased cubic/wurtzite c/w-Ti_0.41_Al_0.57_Y_0.02_N film [26]. By using a Ti_0.46_Al_0.46_Y_0.08_ target, coatings with a predominant wurtzite phase and an AlN and YN mole fraction of x = 0.54 and y = 0.08, respectively, are formed. Hence, these will be referred to as w-Ti_0.38_Al_0.54_Y_0.08_N [26]. When changing the deposition conditions for the Ti_0.49_Al_0.49_Y_0.02_ target from DC sputtering to bipolar pulsed sputtering with small frequencies and long positive pulse durations (see experimental section) the resulting Ti_0.46_Al_0.52_Y_0.02_N coating exhibits a single-phase cubic structure, as described in [27]. Consequently, these films will be referred to as c-Ti_0.46_Al_0.52_Y_0.02_N. The biaxial stresses, obtained by a cantilever beam method [28] of coated Si (100) stripes, are -1.98, -1.81, -0.31, and -0.81 GPa for c-Ti_0.45_Al_0.55_N, c-Ti_0.46_Al_0.52_Y_0.02_N, c/w-Ti_0.41_Al_0.57_Y_0.02_N, and w-Ti_0.38_Al_0.54_Y_0.08_N, respectively.

### 3.1. Simultaneous thermal analyses 

**Figure 1:**
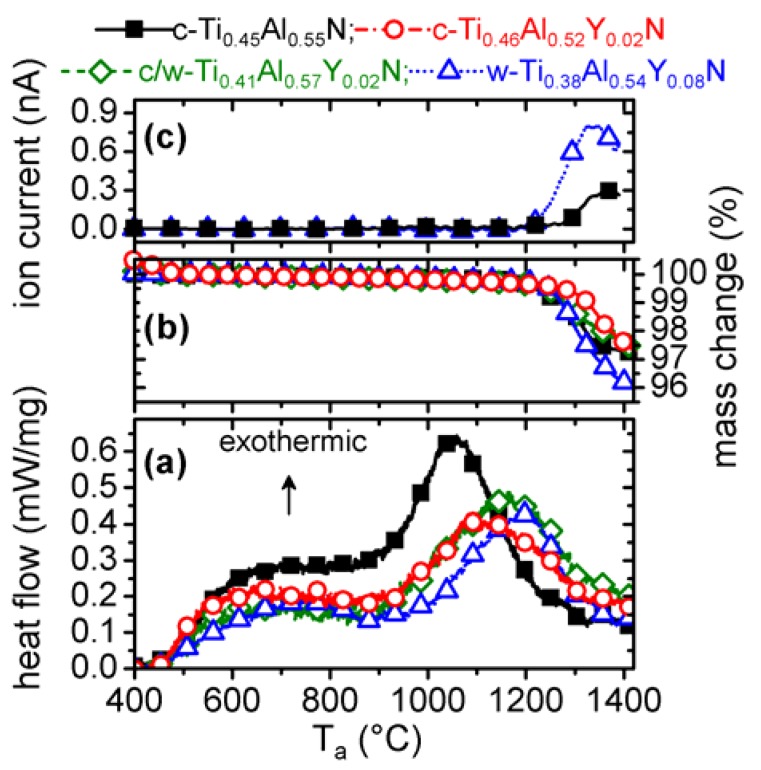
Simultaneous thermal analysis of c-Ti_0.45_Al_0.55_N (0 mol % YN), c-Ti_0.46_Al_0.52_Y_0.02_N (2 mol % YN), binary c/w-Ti_0.41_Al_0.57_Y_0.02_N (2 mol % YN), and single-phase w-Ti_0.38_Al_0.54_Y_0.08_N (8 mol % YN) in He atmosphere up to 1400 °C. **(a)** DSC, **(b)** TGA, and **(c)** MS monitoring of N_2_.

Dynamic annealing of powdered film material during DSC with 20 K/min resulted in a broad exothermic feature in the temperature range of 500 and 900 °C for all coatings investigated (Figure 1a). For T_a_ ≥ 900 °C, pronounced exothermal contributions were detected with peak temperatures increasing from 1052 °C for c-Ti_0.45_Al_0.55_N (0% YN) to 1118 °C for c-Ti_0.46_Al_0.52_Y_0.02_N (2% YN), to 1167 °C for c/w-Ti_0.41_Al_0.57_Y_0.02_N (2% YN), and to 1197 °C for w-Ti_0.38_Al_0.54_Y_0.08_N (8% YN). The TGA and MS measurements suggest a N_2_-release connected mass loss for T_a_ ≥ 1200 °C (Figure 1b and c). The total mass losses during this experiment are ~3% for Y-free c-Ti_1-x_Al_x_N, ~2% for the 2% YN containing films (c-Ti_0.46_Al_0.52_Y_0.02_N and c/w-Ti_0.41_Al_0.57_Y_0.02_N), and ~4% for the 8% YN containing w-Ti_0.38_Al_0.54_Y_0.08_N coating, which correspond to 5.5, 3.8, 3.7 and 7.7 at % N, respectively. Although the EDX measurements performed in the SEM suggest our coatings are slightly over-stoichiometric with nitrogen contents of 51−52 at %, the observed N-release is considerably higher than the 1−2 at % over-stoichiometric nitrogen content. Nitrogen losses exceeding over-stoichiometric content at temperatures between 1000 and 1150 °C for Ti_0.5_Al_0.5_N, V_0.5_Al_0.5_N and Cr_0.5_Al_0.5_N grown in industrial sized sputter devices have also been reported in [31].

Interpretation of the individual DSC features during annealing of the coatings was conducted by structural and phase compositional investigations with XRD and plan-view TEM of free-standing coatings after annealing to various T_a_.

### 3.2. Structure and morphology

#### 3.2.1. Single phase cubic Ti_0.45_Al_0.55_N

**Figure 2:**
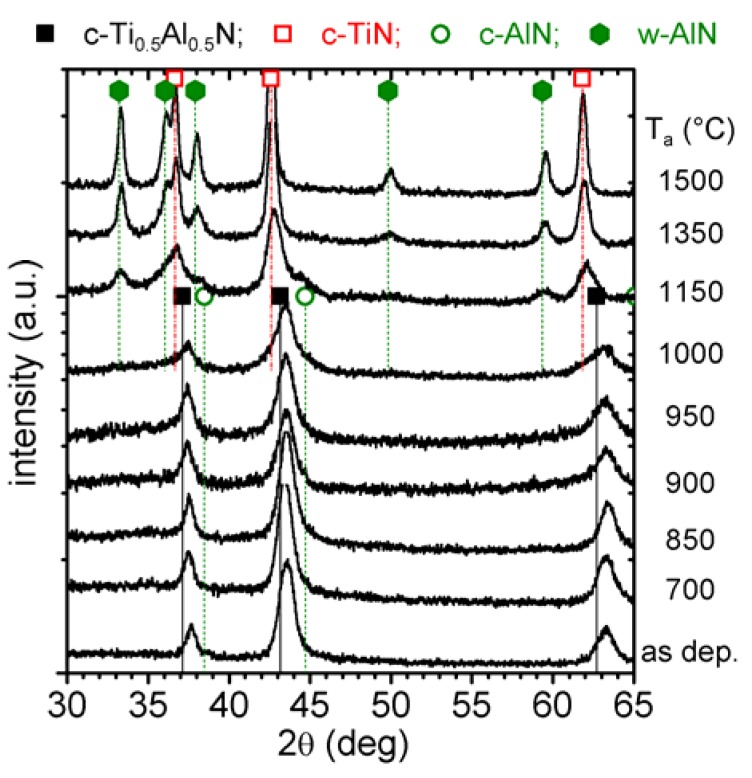
XRD evolution of c-Ti_0.45_Al_0.55_N (0 mol % YN) with annealing temperature T_a_ up to 1500 °C.

XRD patterns of c-Ti_0.45_Al_0.55_N in the as deposited state and after annealing to T_a_ are presented in Figure 2. The as deposited film has a single cubic phase with a lattice parameter *a* of ~4.178 Å [26]. The XRD pattern obtained after annealing to 700 and 850 °C exhibit no major changes to the as deposited state with comparable 2θ positions, intensities, and widths of the XRD peaks. Therefore, the exothermic DSC feature in the temperature range 500−850 °C is attributed to recovery processes of deposition-induced defects, which generally result in stress relaxation as described in [9,11]. The XRD pattern of the 900 °C annealed coating reveals a decrease in peak intensities but an increase in peak broadening of the cubic Ti_1-x_Al_x_N reflexes. This indicates grain refinement and probably also an increase in microstresses due to spinodal decomposition [16,32].

In the Ti_1-x_Al_x_N system a characteristic sign for spinodal decomposition is a shoulder formation on both sides—*i.e.,* higher and lower diffraction angles—of the cubic matrix XRD reflections. This can especially be observed in the 2θ range 41–47 deg (around the (200) reflex), since here the individual standard positions of c-TiN, c-AlN, and w-AlN do not interfere, see Figure 2. After annealing to 900 °C, the XRD patterns exhibit these shoulders on both sides of the cubic reflexes and hence they provide a clear evidence for spinodal decomposition of the cubic Ti_0.45_Al_0.55_N solid-solution, as proven by various calculations and experiments [6,11,16,32]. A broad x-ray diffraction response in the 2θ range 32−36 deg indicates the formation of the wurtzite phases.

When the coating is further annealed, the cubic solid-solution matrix XRD reflexes decrease in intensity and shift to lower 2θ diffraction angles (which can especially be seen for the peak at ~63 deg) for temperatures > 950 °C. Simultaneously, the shoulders on both sides of the cubic matrix peak, indicating the formation of cubic Al- and Ti-rich domains, become more pronounced. The XRD pattern of the coatings after annealing at 1000 °C clearly shows this shoulder formation, *i.e.,* the spinodal decomposition.

After annealing the coating to 1150 °C, strong reflexes for c-TiN and w-AlN can be detected by XRD at the expense of the cubic solid-solution matrix. In addition, the XRD pattern exhibits evidence for remaining c-AlN phases at this annealing stage. The XRD pattern of the 1350 and 1500 °C annealed samples reveal a fully decomposed microstructure, where the individual peaks fit to c-TiN and w-AlN, only traces of c-AlN can be detected. In addition, the XRD peaks sharpened, indicating the growth of the recrystallized grains and change in texture.

**Figure 3:**
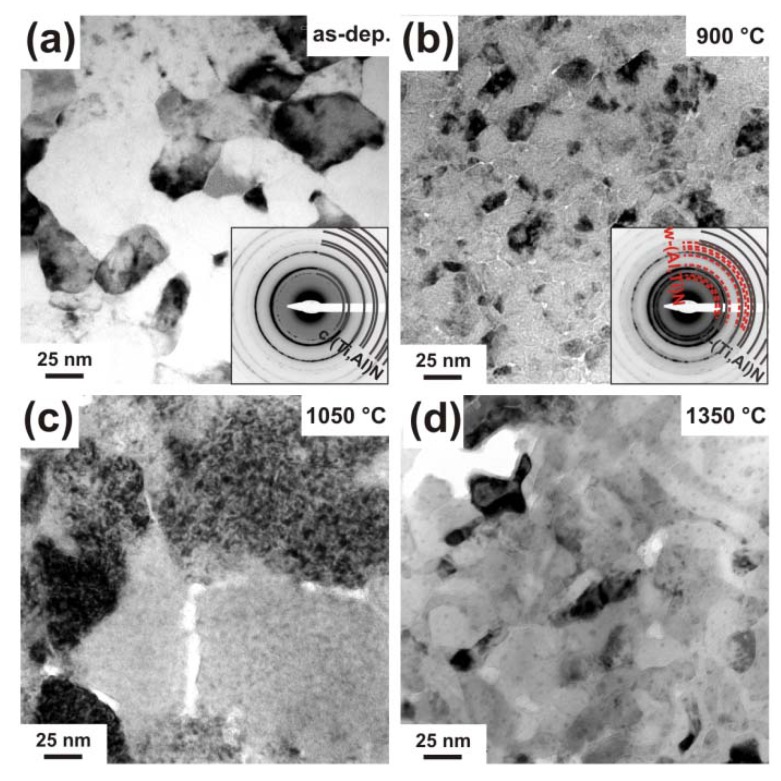
Plan view TEM micrographs of c-Ti_0.45_Al_0.55_N in the (a) as deposited state and after annealing to (b) 900 °C, (c) 1050 °C, and (d) 1350 °C. The small insets in (a) and (b) show their corresponding SAED patterns.

The morphological changes in c-Ti_0.45_Al_0.55_N due to the annealing treatment and the observed structural changes were investigated by TEM, see Figure 3. The as deposited film (Figure 3a) has a dense columnar microstructure (as proven by earlier studies, see [26,27]) with relatively large column diameters of ~100 nm, and the corresponding selected area electron diffraction pattern (SAED, inset in Figure 3a) confirms the single-phase cubic structure observed by XRD. TEM investigations of the coating after annealing to 900 °C, Figure 3b, suggest an average grain size of ~30 nm. In a recent investigation on the thermal decomposition of Ti_1-x_Al_x_N by atom probe tomography, clustering of Al-rich and Al-depleted Ti_1-x_Al_x_N solid-solution areas within the c-Ti_1-x_Al_x_N matrix grains was observed after annealing to 900 °C [19]. In addition to these results, we showed that the spinodal decomposition to form alternating Al- and Ti-rich domains can also be connected with the formation of an incoherent, wurtzite AlN-rich phase at the grain boundary.

The TEM investigation of a sample annealed to 1050 °C (Figure 3c) reveals a microstructure composed of domains with ~150 nm in diameter and a grain boundary phase with >10 nm in thickness. The recrystallized coating structure after annealing at 1350 °C is shown in the TEM plan-view micrograph in Figure 3d. The image exhibits an interlinked network of >100 nm sized elongated, vermicular shaped grains and <10 nm sized precipitates within the domains. Such interconnected microstructures are typical for spinodally decomposed materials and are described in literature theoretically and experimentally [33,34,35]. A corresponding interlinked three-dimensional network has recently been presented by atom probe investigations of 1350 °C annealed Ti_1-x_Al_x_N [19]. The decrease in grain size from the 1050 °C annealed sample to the 1350 °C annealed sample is due to the formation of new phases, compare Figure 2.

**Figure 4:**
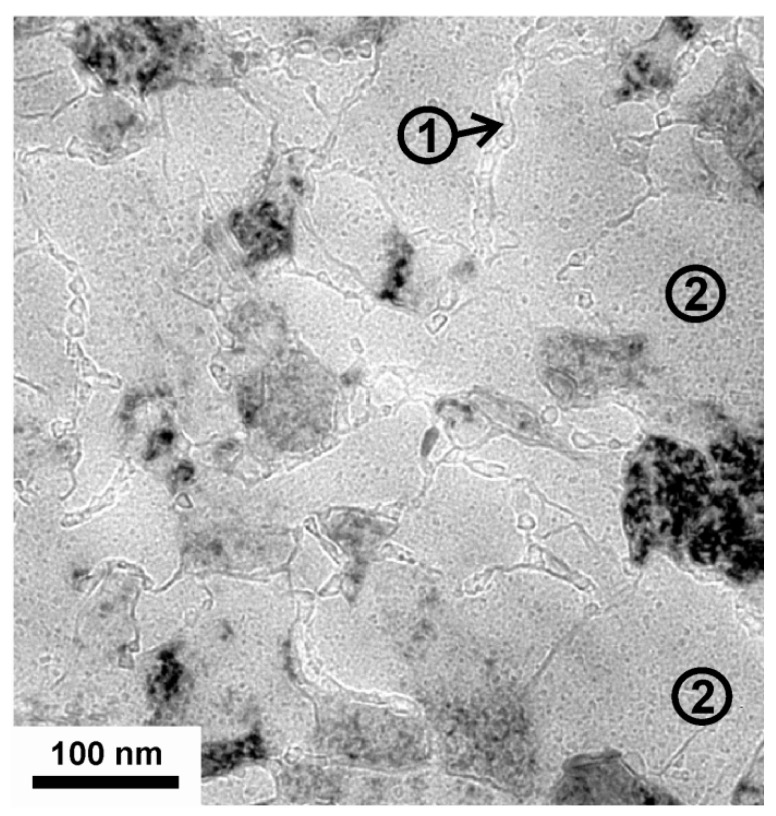
Over-focused plan view TEM micrograph of c-Ti_0.45_Al_0.55_N after annealing to 1050 °C taken at a lower magnification than in Figure 3. The grain boundary phase is indexed with (1) and the formation of nm-size precipitates within the grains are indexed with (2).

Detailed plan-view TEM investigations of the 1050 °C annealed coatings are presented in over-focus condition in Figure 4. Along with the large grains, which hint a still existing columnar microstructure, the grain boundary phase is visible, see the area labeled with (1) in Figure 4. Also, precipitates with average sizes of ~7 nm diameter within the large domains can be identified, see e.g., the areas labelled with (2) in Figure 4. Domain sizes around 6 nm in diameter of 1050 °C annealed Ti_0.5_Al_0.5_N coatings were also detected by small angle neutron scattering [16].

Generally, spinodal decomposition of the cubic solid-solution Ti_1-x_Al_x_N can be expected at temperatures above 850 °C [9,11,20,22] which allow for the required kinetic conditions. The spinodal decomposition is a spontaneous process where a cluster or concentration fluctuation amplifies to the equilibrium concentration through diffusion up the concentration gradient [36], when the kinetic requirements allow for it. Therefore, spinodal decomposition will take place within the domains or grains of a solid-solution. In recent years, the effect of grain boundaries and inner surface on spinodal decomposition was investigated and it was found that these surfaces can also stimulate the appearance of concentration waves. Such surface-initiated or directed spinodal decomposition leads to domains repeating the grain boundary geometry [34,35,37].

With the above described changes in structure and morphology the detected features during DSC (Figure 1a) can be interpreted. Starting from temperatures slightly above the deposition temperature, recovery processes are responsible for the detected DSC signal, where also the formation of w-AlN for T_a_ ≥ 700 °C and cubic Ti- and Al-rich domains contribute. The pronounced exothermic feature between 910 and 1300 °C includes ongoing spinodal decomposition and formation of w-AlN. For T_a_ ≥ 1150 °C, the Ti_0.45_Al_0.55_N thin film is largely decomposed into the stable constituents c-TiN and w-AlN. With increasing temperature T_a_ the fraction of c-AlN decreases. For T_a_ ≥ 1350 °C, recrystallization and coarsening are responsible for the detected DSC features. These exothermal processes may be superimposed by endothermic contributions resulting from nitrogen release observed for T_a_ ≥ 1140 °C (Figure 1b and c).

In general, the decomposition process found for the herein investigated Ti_0.45_Al_0.55_N differs by the early formation of the wurtzite phase from the decomposition route described in [9,11] for single phase cubic Ti_1-x_Al_x_N. The decomposition reactions, especially the transformation of c-AlN to stable w-AlN, occurs at lower temperatures. Nevertheless, the overall energy input for the decomposition, *i.e.,* the integrated area under the entire DSC curve, is 35.3 kJ mol^-1^ comparable to the 36.2 kJ mol^-1^ reported in [16] for the decomposition of Ti_0.5_Al_0.5_N.

#### 3.2.2. Single phase cubic Ti_0.46_Al_0.52_Y_0.02_N

**Figure 5:**
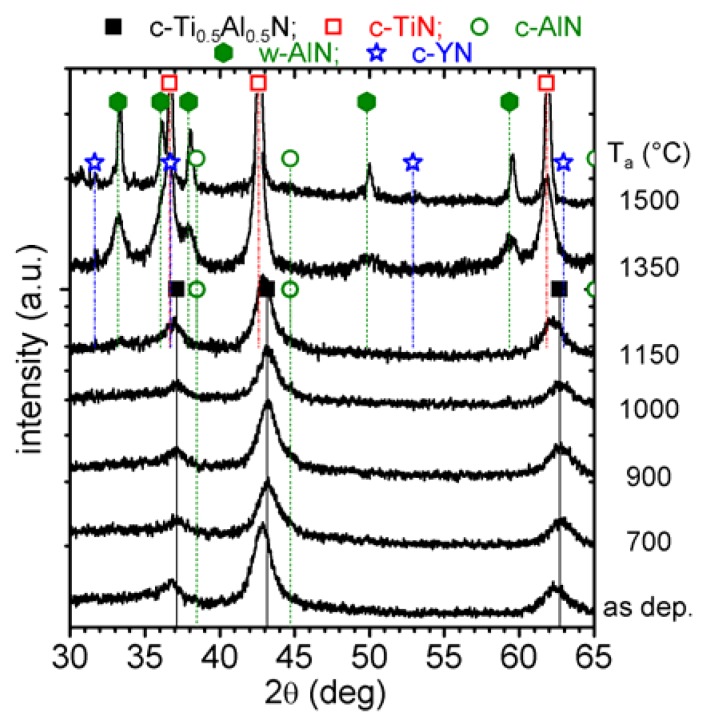
XRD evolution of single-phase c-Ti_0.46_Al_0.52_Y_0.02_N with annealing temperature T_a_ up to 1500 °C.

The XRD patterns of powdered 2 mol % YN containing Ti_1-x_Al_x_N (removed from their low alloy steel substrates) in their as deposited state and after annealing at T_a_ are presented in Figure 5. These films are prepared by reactive pulsed DC magnetron sputtering using a Ti_0.49_Al_0.49_Y_0.02_ target, see experimental section, and exhibit a cubic stabilized structure with a chemical composition of c-Ti_0.46_Al_0.52_Y_0.02_N [27]. The strong (200) reflex as well as the smaller (111) reflex for a cubic phase indicate solid-solution.

Upon annealing to 700, 900, and 1000 °C, decreasing intensities of these cubic solid-solution matrix-reflexes are observed. The shoulder-formation on both sides of these reflexes, characteristic for cubic Al- and Ti-rich domains (as these would have smaller and larger lattice parameters, respectively) can already be detected for T_a_ = 700 °C, *i.e.,* ~200 °C earlier than for the c-Ti_0.45_Al_0.55_N film, compare Figure 2. The XRD pattern of c-Ti_0.46_Al_0.52_Y_0.02_N annealed at 1150 °C still exhibits the presence of cubic solid-solution matrix, along with c-TiN, small fractions of c-AlN, and w-AlN. The formation of c-YN precipitates is suggested by a small XRD response at ~31.6 deg. The observed retarded decomposition of the c-Ti_0.46_Al_0.52_Y_0.02_N solid-solution as compared to c-Ti_0.45_Al_0.55_N, where at 1150 °C the XRD peaks are already close to the positions of the stable constituents, is attributed to Y induced effects as observed also for Cr-Al-Y-N [18,38]. After annealing at T_a_ = 1350 °C, the XRD pattern indicates only reflexes that can be assigned to the stable phases c-TiN, c-YN, and w-AlN, with only minor indications for c-AlN (Figure 5). When the film is further annealed to 1500 °C, only pronounced high intensity XRD peaks can be detected with 2θ−values corresponding to c-TiN, c-YN, and w-AlN indicating recrystallization and coarsening of the decomposed material.

**Figure 6:**
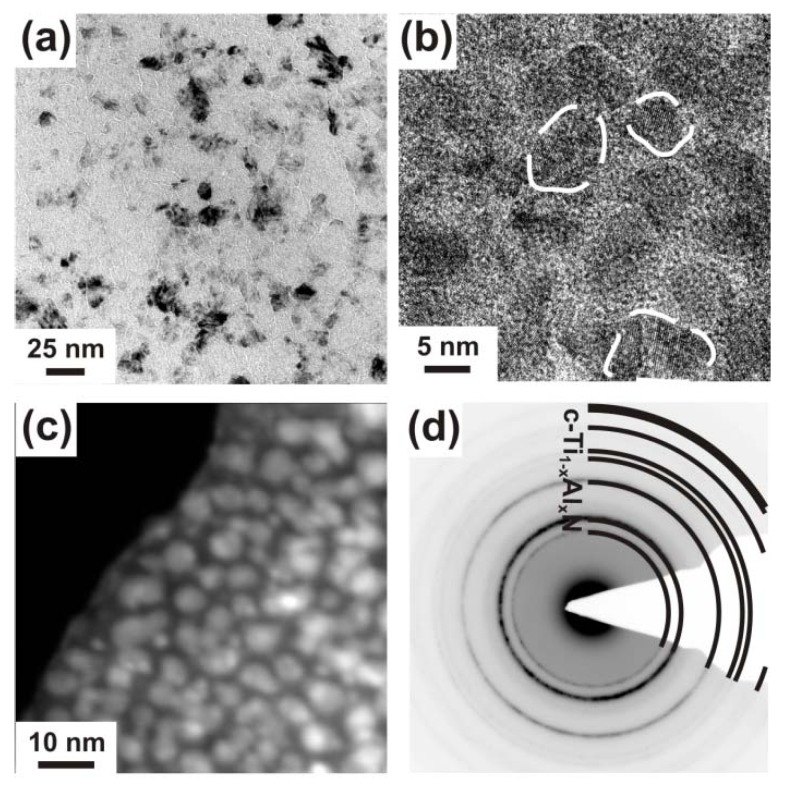
Plan view TEM micrographs of the single-phase c-Ti_0.46_Al_0.52_Y_0.02_N taken in (a) bright-field low magnification, (b) HRTEM and (c) HAADF STEM mode. The crystallite areas are highlighted by the dashed-line in the HRTEM image. (d) SAED pattern showing only cubic solid-solution Ti_1-x_Al_x_N reflexes.

The morphological evolution with annealing temperature of this c-Ti_0.46_Al_0.52_Y_0.02_N coating is investigated by plan view TEM, see Figure 6.

In the as deposited state the coating exhibits a fine-grained microstructure with domain sizes of <10 nm, much smaller as compared to the ~100 nm of the Y-free c-Ti_0.45_Al_0.55_N film (compare Figure 3 and Figure 6). The grain boundaries of the c-Ti_0.46_Al_0.52_Y_0.02_N coating are more pronounced, as a bright-contrast tissue-phase can be identified in the bright-field image (Figure 6a). In the HAADF image the tissue phase appears dark (Figure 6c), indicating a lower average atomic number compared to the grains. The HRTEM and HAADF images (Figure 6b and c, respectively), exhibit 5−10 nm sized grains (cross-section of the coating-columns, highlighted by the dashed line) encapsulated by the grain boundary phase with thicknesses up to a few nm. Lattice fringes can only be detected within the grains. The SAED pattern (Figure 6d), taken over an area covering several grains as well as the tissue phase, shows only diffraction-rings for a cubic solid-solution with a lattice parameter of ~4.178 Å, comparable to that obtained by XRD. Based on these investigations, we assume that the grain-boundary phase has a low short-range order structure resulting in an electron (SAED) and X-ray (XRD) amorphous response.

When performing electron energy-loss spectroscopy (EELS) and EDX in the TEM on the samples, an increased Al content can be detected within the grain-boundary phase with respect to the encapsulated grains. The contents for Ti and Y are evenly distributed within the investigated areas of the as deposited films. As the chemical composition of this film is close to the border line between cubic and wurtzite phase-fields, see [26,27], depending on the deposition conditions and the resulting kinetics (which influence the phase-field ranges), either the cubic or the wurtzite phase can be favored, or both phases could co-exist. Such processes are fundamentally described in [39,40], and competitive-growth resulting structures are reported in literature for a large variety of different coating systems, ranging from metallic films to nanocomposites, e.g., based on transition metal nitrides or borides [41,42,43,44].

**Figure 7:**
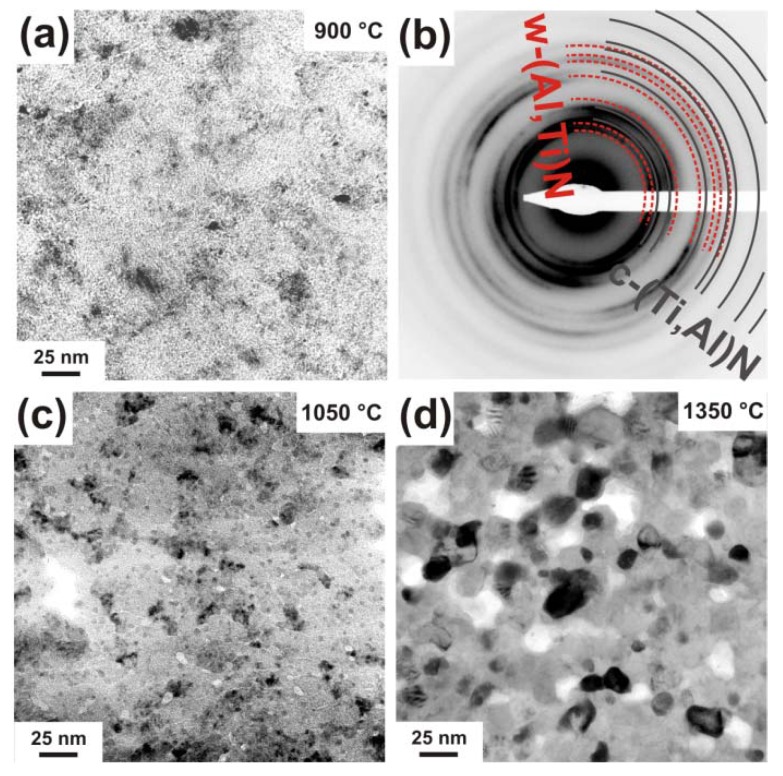
TEM plan view micrograph of the single-phase c-Ti_0.46_Al_0.52_Y_0.02_N after annealing to (a) 900 °C, (c) 1050 °C, and (d) 1350 °C. (b) SAED pattern for the 900 °C annealed sample.

Corresponding to the results obtained for c-Ti_0.45_Al_0.55_N, also the c-Ti_0.46_Al_0.52_Y_0.02_N film reveals a decreasing grain size with increasing T_a_ to 900 °C (compare Figure 6a and Figure 7a). The corresponding SAED pattern, Figure 7b, suggests cubic as well as wurtzite phase contributions.

After annealing to 1050 °C, Figure 7c, the coating exhibits ~20 nm sized domains, surrounded by a bright-contrast phase with diameters between 4.5 and 12 nm. These bright areas could be identified as holes. As for these sample areas, the SAED pattern (not shown here) indicate a decreased wurtzite phase content (as compared to the lower annealing temperatures) the holes might be related to former w-AlN areas. As the formation of wurtzite AlN from the cubic Ti_1-x_Al_x_N phase is connected to a huge volume increase (w-AlN has a ~26% higher specific volume as its metastable cubic counterpart [6]) these areas might break during the TEM sample preparation.

Within the domains, small ~3.5 nm sized precipitations can be identified, similar to the 1050 °C annealed Y-free c-Ti_0.45_Al_0.55_N film. Whereas the latter exhibits an interconnected network of >100 nm sized grains after annealing at 1350 °C (see Figure 3), the microstructure of the c-Ti_0.46_Al_0.52_Y_0.02_N film annealed at the same temperature is composed of only ~25 nm globular grains (Figure 7d). This grain refinement effect can be attributed to Y-induced effects and the additional precipitation of c-YN.

Based on these TEM and XRD investigations, the DSC measurements of the c-Ti_0.46_Al_0.52_Y_0.02_N sample, shown in Figure 1a, can be interpreted with respect to the findings for the Y-free c-Ti_0.45_Al_0.55_N sample. Similar to the Y-free film, the first exothermic DSC feature starting at ~500 °C includes recovery processes, spinodal decomposition, as well as formation of w-AlN. Our results suggest that the smaller grain size and the high-Al containing boundary phase with a lower density promote the formation of w-AlN. Cubic AlN can already be detected after annealing at ~700 °C, hence at lower temperatures as for the Y-free film, compare Figure 2 and Figure 5. In contrast to these results, the development of the w-AlN phase is retarded to higher temperatures. This is also represented in the DSC curve with an increase in peak temperature from 1052 °C for c-Ti_0.45_Al_0.55_N to 1118 °C for c-Ti_0.46_Al_0.52_Y_0.02_N (see Figure 1a). Consequently, the onset of mass loss is also shifted by 60 °C from 1140 °C for c-Ti_0.45_Al_0.55_N to ~1200 °C for c-Ti_0.46_Al_0.52_Y_0.02_N (Figure 1b). Yttrium retards the decomposition of the solid-solution as well as the transformation of c-AlN to w-AlN. The early onset of the formation of w-AlN is related to its development from the grain boundary phase. It is envisioned that a further optimization of the deposition conditions, where the amount of the grain boundary phase can be reduced and the cubic phase can further be stabilized, will result in a retarded formation of w-AlN, corresponding to the results obtained for Cr-Al-Y-N [18].

#### 3.2.3. Binary phase cubic/wurtzite Ti_0.41_Al_0.57_Y_0.02_N

**Figure 8:**
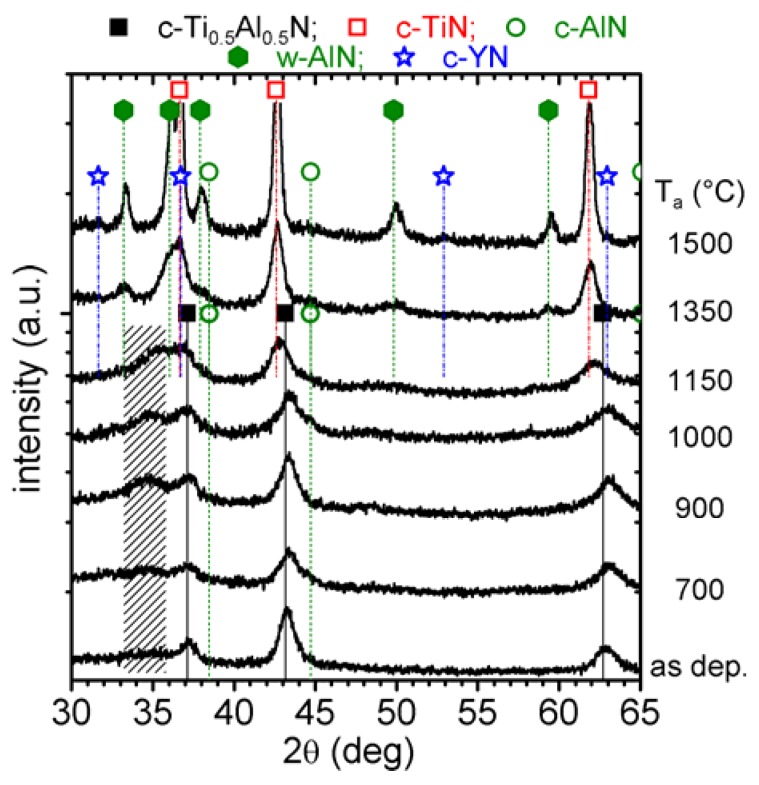
XRD evolution of binary c/w-Ti_0.41_Al_0.57_Y_0.02_N with annealing temperature T_a_ up to 1500 °C.

The XRD pattern of the as deposited 2 mol % YN containing film, prepared by reactive magnetron DC sputtering of a Ti_0.49_Al_0.49_Y_0.02_ target, suggests a binary phase structure composed of cubic and wurtzite (hatched area) solid-solution Ti_1-x-y_Al_x_Y_y_N phases, see Figure 8. While the wurtzite phase fraction results in a relatively low-intensity reflection (2θ range between 32 and 37 deg) in the as deposited state, this intensity increases with increasing T_a_ to 1150 °C.

Corresponding to the cubic stabilized c-Ti_0.46_Al_0.52_Y_0.02_N coating, also this binary c/w-Ti_0.41_Al_0.57_Y_0.02_N coating exhibits increasing c-AlN reflexes (2θ ~44.7 deg) upon annealing to 700 °C, suggesting the onset of spinodal decomposition at relatively low temperatures. Annealing the coating to 1000 and 1150 °C, causes an increasing intensity for the XRD reflexes attributed to the wurtzite solid-solution at 2θ ~34 deg as well as the c-AlN at 2θ ~44.7 deg. The c-Ti_1-x-y_Al_x_Y_y_N peaks shift to lower diffraction angles, suggesting an increase in lattice constant and hence a depletion in AlN or enrichment in TiN of the cubic solid-solution. After annealing to 1350 °C the coating is mainly composed of phases with lattice constants close to their standard values, hence the XRD peaks match the 2θ positions for c-TiN and w-AlN, see Figure 8. Nevertheless, the broad reflexes still suggest small grain sizes, similar to the results obtained for c-Ti_0.46_Al_0.52_Y_0.02_N, and indicate retarded decomposition and transformation as compared to the Y-free c-Ti_0.45_Al_0.55_N film. With increasing the temperature to 1500 °C, the individual grains grow (increased sharpness of the respective XRD reflexes), and also c-YN can clearly be detected (2θ ~31.5 and 52.5 deg).

**Figure 9:**
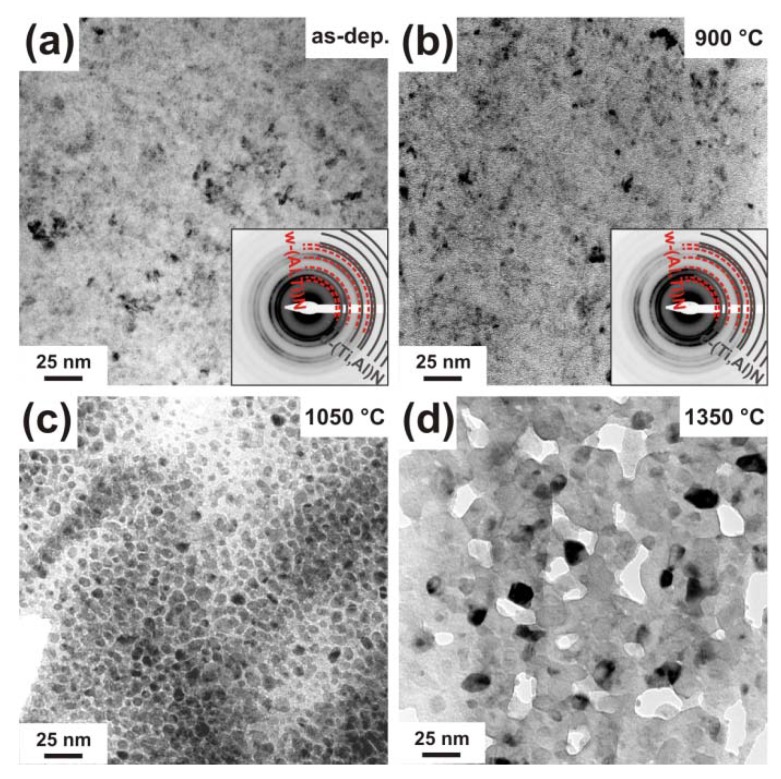
Plan view TEM micrographs of binary c/w-Ti_0.41_Al_0.57_Y_0.02_N in the (a) as deposited state, and after annealing to (b) 900 °C, (c) 1050 °C, and (d) 1350 °C. The small insets in (a) and (b) are the corresponding SAED patterns.

Plan view TEM investigations of this binary phased c/w-Ti_0.41_Al_0.57_Y_0.02_N coating exhibit a fine grained morphology in the as deposited state, see Figure 9a.

In [27] we investigated this film by cross-sectional TEM and found a tilted columnar microstructure with column diameters at the coating surface below 40 nm in the as deposited state. The plan view investigations, here, suggest a grain size below 20 nm. The SAED pattern of the as deposited film (inset in Figure 9a) clearly shows the co-existence of cubic and wurtzite phases. After annealing to 900 °C, the TEM plan view micrographs as well as the corresponding SAED pattern show no major changes as compared to the as deposited condition (Figure 9b). After annealing to 1050 °C, the coating consists of grains with diameters in the range 5–15 nm, surrounded by a bright-contrast tissue phase, see Figure 9c. Annealing the coating further to 1350 °C results in the formation of a coarsened (but still small sized), globular grained microstructure, see Figure 9d.

Corresponding to the findings for the c-Ti_0.45_Al_0.55_N and the c-Ti_0.46_Al_0.52_Y_0.02_N coating also for this binary phased c/w-Ti_0.41_Al_0.57_Y_0.02_N coating relaxation and recovery effects are responsible for the DSC features between ~430 °C and the onset temperature of the large exothermic peak at ~920 °C. The latter covers the processes of decomposition, recrystallization and grain growth. In total the energy released of this c/w-Ti_0.41_Al_0.57_Y_0.02_N coating during annealing to 1500 °C is ~19.22 kJ mol^-1^.

#### 3.2.4. Single phase wurtzite Ti_0.38_Al_0.54_Y_0.08_N

**Figure 10:**
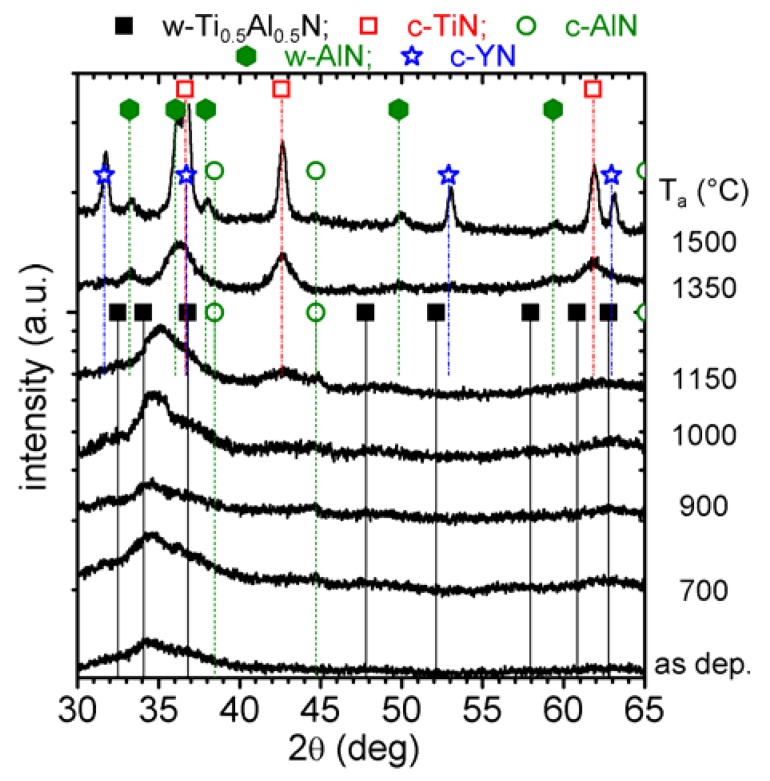
XRD evolution of single-phase w-Ti_0.38_Al_0.54_Y_0.08_N with annealing temperature T_a_ up to 1500 °C.

The XRD pattern of the as deposited 8 mol % YN containing Ti_0.38_Al_0.54_Y_0.08_N film exhibits a solid-solution wurtzite structure with lattice parameters of a ~3.205 Å and c ~5.220 Å [26], see Figure 10. With increasing temperature to 1000 °C, the XRD patterns suggest only minor changes in structure. The increased XRD reflex intensities indicate only an increased crystallinity. When the coatings are annealed to 1150 °C, new phases can be detected which can be assigned to c-TiN (2θ ~43 deg). Hence, the solid-solution wurtzite structure decomposes to form TiN precipitates.

After annealing to 1350 °C, the XRD reflexes can be attributed to the stable phases c-TiN, w-AlN, and c-YN, although the reflexes are still broad. Hence, the grain size is still small, even after annealing to 1350 °C. With a further increase in T_a_ to 1500 °C, the XRD peaks are clearly separated and suggest further recrystallization and grain growth. Here, also the c-YN reflexes can clearly be identified.

**Figure 11:**
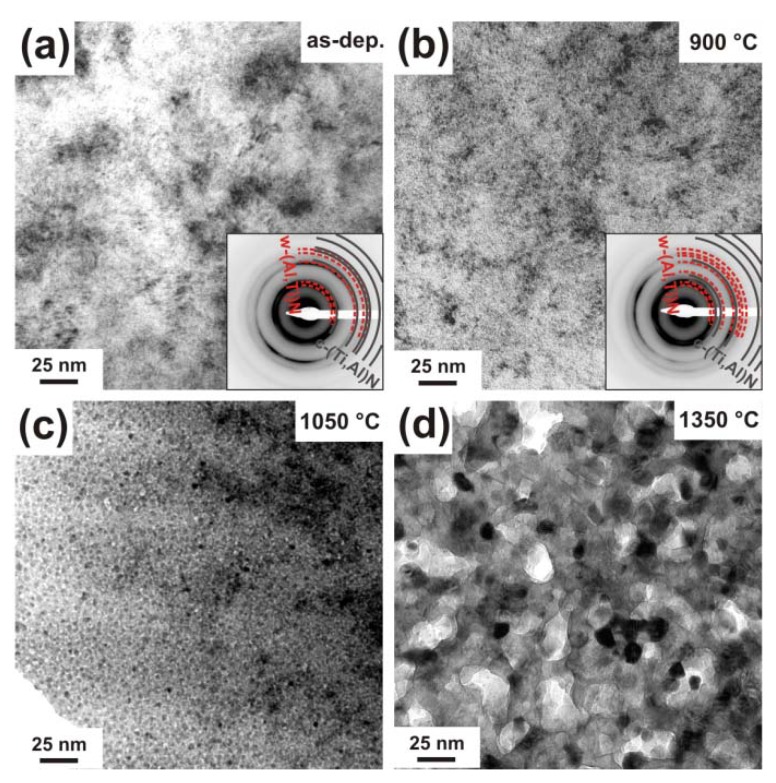
Plan view TEM micrographs of w-Ti_0.38_Al_0.54_Y_0.08_N in the (a) as deposited state and after annealing at (b) 900 °C, (c) 1050 °C, and (d) 1350 °C. The small insets in (a) and (b) are the corresponding SAED patterns.

The plan view TEM investigations of the as deposited w-Ti_0.38_Al_0.54_Y_0.08_N coating exhibit a very small grain size, see Figure 11a, as suggested by XRD. The corresponding SAED pattern indicates mainly diffraction rings due to a wurtzite phase and almost no indication for a cubic phase (inset in Figure 11a). The TEM plan view as well as the corresponding SAED investigations exhibit no major changes due to an annealing treatment to 900 °C (see Figure 11b), as compared to the as deposited film. This again is in agreement with the XRD results. Annealing the film to 1050 °C causes the formation of a clear crystalline grain structure with grain sizes around 3–5 nm, see Figure 11c. Based on the XRD results, this is attributed to the decomposition of the wurtzite phase solid-solution and the precipitation of c-TiN. These grains coarsen during further annealing (Figure 11d). 

The small changes in XRD and TEM response between the as deposited and up to 1000 °C annealed w-Ti_0.38_Al_0.54_Y_0.08_N samples are in agreement with the small DSC feature between 400 and 1000 °C. The peak temperature with ~1200 °C for this coating is the highest compared to the films probed here, as already identified by XRD and TEM investigations. Also, the onset temperature for mass loss is with 1210 °C highest for this high Y-containing film. The overall energy released during DSC is ~19.15 kJ mol^-1^ and comparable to that of the c/w-Ti_0.41_Al_0.57_Y_0.02_N with 19.22 kJ mol^-1^ and c-Ti_0.46_Al_0.52_Y_0.02_N with 20.49 kJ mol^-1^.

### 3.3. Hardness development

**Figure 12:**
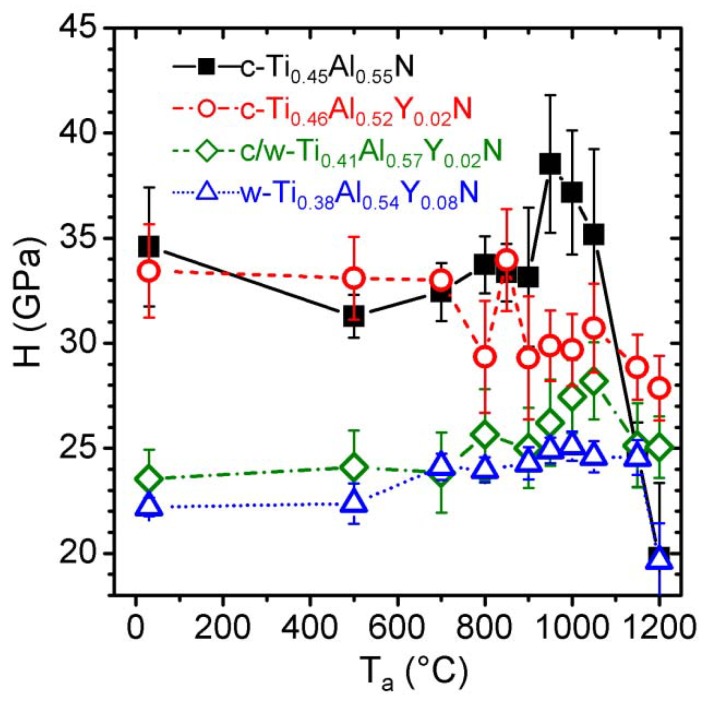
Hardness evolution with post-deposition annealing in He atmosphere up to 1200 °C of c-Ti_0.45_Al_0.55_N (0 mol % YN), c-Ti_0.46_Al_0.52_Y_0.02_N (2 mol % YN), binary c/w-Ti_0.41_Al_0.57_Y_0.02_N (2 mol % YN), and single-phase w-Ti_0.38_Al_0.54_Y_0.08_N (8 mol % YN).

The hardness evolution of the c-Ti_0.45_Al_0.55_N, c-Ti_0.46_Al_0.52_Y_0.02_N, c/w-Ti_0.41_Al_0.57_Y_0.02_N, and w-Ti_0.38_Al_0.54_Y_0.08_N coatings with annealing temperatures up to 1200 °C is presented in Figure 12. Due to coating flaking no hardness data could be evaluated for higher annealing temperatures. Due to the different microstructures of the various as deposited coatings their hardness varies tremendously. While the single-phase c-Ti_0.45_Al_0.55_N and c-Ti_0.46_Al_0.52_Y_0.02_N films have high hardnesses of 34.6 ± 2.8 and 33.4 ± 2.3 GPa, the binary c/w-Ti_0.41_Al_0.57_Y_0.02_N film has only 23.5 ± 1.4 GPa, and the nearly single phase wurtzite w-Ti_0.38_Al_0.54_Y_0.08_N coating has the lowest hardness with 22.2 ± 0.4 GPa.

Due to the annealing induced recovery, the hardness of c-Ti_0.45_Al_0.55_N decreases slightly to ~32 GPa at 500 °C. With a further increase in temperature, the hardness increases due to the ongoing spinodal decomposition and the formation of various obstacles for the dislocation movement. The peak-hardness of 38.5 ± 3.2 GPa is obtained after annealing to 950 °C, see Figure 12. At higher temperatures coarsening of the individual domains as well as the ongoing formation of w-AlN and recrystallization lead to a decrease in hardness to 19.8 ± 3.5 GPa at 1200 °C.

The single-phase c-Ti_0.46_Al_0.52_Y_0.02_N film has an H over T_a_ curve comparable to the Y-free coating up to temperatures of ~700−850 °C. The smaller grain size as compared to the c-Ti_0.45_Al_0.55_N coating may explain the less pronounced effect of the spinodal decomposition on the hardness at temperatures above 850 °C. The formation of cubic domains, which takes place within the grains and at grain boundaries, might result in lower coherency strains for small grain-sized samples. Consequently, fewer additional obstacles for dislocation movement are provided. Hence, for this c-Ti_0.46_Al_0.52_Y_0.02_N coating no hardness increase upon annealing above 900 °C is obtained but a small reduction in H to ~30 GPa occurs. Nevertheless, the hardness remains almost constant at ~30 GPa for T_a_ between 900 and 1050 °C, which is in excellent agreement to the small changes in structure and morphology for annealing temperatures up to 1000 °C. Ongoing decomposition of the cubic solid-solution and formation of w-AlN as well as transformation of c-AlN to w-AlN at T_a_ > 1050 °C results in a hardness decrease. But these coatings exhibit the highest hardness values with 28.9 ± 1.5 GPa at 1150 °C and 27.9 ± 1.5 GPa at 1200 °C of the coatings investigated.

The binary c/w-Ti_0.41_Al_0.57_Y_0.02_N coating has an almost constant hardness of ~24 GPa for temperatures up 700 °C. Crystallization of the Al-rich boundary phases as well as the formation of c-AlN domains result in a slight increase in hardness to 25.0 ± 1.9 GPa at 900 °C. The decomposition of the wurtzite phase solid-solution to form c-TiN and the formation of a nano-composite lead to a further increase in H to 28.2 ± 1.8 GPa at 1050 °C. At higher temperatures, the ongoing w-AlN phase formation causes a reduction in H to 25.1 ± 1.5 GPa at 1200 °C, which is still a higher hardness than for the as deposited condition with 23.5 ± 1.4 GPa and also exceeds the hardness of c-Ti_0.45_Al_0.55_N at 1200 °C.

Due to the pronounced wurtzite phase content for w-Ti_0.38_Al_0.54_Y_0.08_N coatings up to T_a_ ~1150 °C, their hardness is the lowest of the coatings investigated. A small increase form 22.3 ± 0.9 GPa at 500 °C to 24.6± 0.8 GPa at 1150 °C can be related to the decomposition of the wurtzite phase solid-solution to form c-TiN and c-YN precipitates, see Figure 11 and Figure 12.

## 4. Summary and Conclusions

Reactive DC magnetron sputtering of powder metallurgically prepared Ti_0.50_Al_0.50_, Ti_0.49_Al_0.49_Y_0.02_, and Ti_0.46_Al_0.46_Y_0.08_ targets result in the formation of single-phase c-Ti_0.45_Al_0.55_N, binary c/w-Ti_0.41_Al_0.57_Y_0.02_N and single-phase w-Ti_0.38_Al_0.54_Y_0.08_N coatings. By applying pulsed DC to the Ti_0.49_Al_0.49_Y_0.02_ target, instead of DC, the coating can be stabilized in its cubic metastable state, as thereby the deposition conditions change and the Al content of the film decreases to c-Ti_0.46_Al_0.52_Y_0.02_N. This allows for detailed investigations of the thermal stability of Ti_1-x-y_Al_x_Y_y_N coatings as a function of their Y-content as well as their microstructure and morphology. Differential scanning calorimetry in combination with thermo gravimetric analyses and mass spectroscopy in He atmosphere reveal increasing thermal stability with increasing Y content from y = 0.0 to 0.08 as the peak temperature of the major exothermic feature increases from 1052 to 1197 °C. XRD in combination with TEM and HRTEM investigations suggest the highest contribution of spinodal decomposition to the overall transformation for the Y-free film c-Ti_0.45_Al_0.55_N, which exhibit also the largest grain size (column diameter) of ~100 nm in the as deposited state. Consequently, this coating shows the most pronounced hardness increase from ~35 to 38 GPa when annealed to 950 °C. The cubic stabilized and Y-alloyed coating c-Ti_0.46_Al_0.52_Y_0.02_N has an as deposited grain size of ~10 nm and hardness of 33 GPa. HRTEM, EDS and EELS suggest that the few nm thin grain boundary phase has a comparable Ti and Y content, but higher Al content as the encapsulated grains. These areas promote the formation of AlN upon annealing to higher temperatures. Consequently, cubic as well as wurtzite phase AlN can be observed already after annealing at 700 °C. Nevertheless, the overall decomposition of the metastable cubic solid-solution Ti_1-x-y_Al_x_Y_y_N is retarded with respect to the Y-free coating. This c-Ti_0.46_Al_0.52_Y_0.02_N coating has the highest hardness at T_a_ ≥ 1000 °C of the coatings investigated with ~29 and 28 GPa for T_a_ = 1150 and 1200 °C, respectively.

Due to the wurtzite phase fraction of the c/w-Ti_0.41_Al_0.57_Y_0.02_N and w-Ti_0.38_Al_0.54_Y_0.08_N coatings, their hardness is with ~24 and 22 GPa, the smallest in the as deposited state. But due to the Y-content these coatings exhibit a high thermal stability, where annealing related microstructural changes and the formation of Al- and Ti-rich domains for c/w-Ti_0.41_Al_0.57_Y_0.02_N result in an hardness increase to ~28 GPa at T_a_ = 1050 °C. Almost no c-AlN formation could be detected during the annealing treatment of the almost single phase w-Ti_0.38_Al_0.54_Y_0.08_N coatings. Consequently, their hardness only increases to ~25 GPa upon annealing to T_a_ between 700 and 1150 °C, caused by the precipitation of c-TiN and c-YN.

Based on our results, we can conclude that Y effectively retards the decomposition process of supersaturated phases. Furthermore, it is envisioned that an optimization of the deposition conditions, where the amount of the grain boundary phase (which can act as nucleation sites for AlN) can be reduced and the cubic phase can further be stabilized, will result in a retarded formation of w-AlN. Thereby, the hardness reduction due to the promoted formation of w-AlN can be shifted to higher temperatures.
